# *DNMT3A* Mutations in Patients with Acute Myeloid Leukemia in South Brazil

**DOI:** 10.1155/2012/697691

**Published:** 2012-11-08

**Authors:** Annelise Pezzi, Lauro Moraes, Vanessa Valim, Bruna Amorin, Gabriela Melchiades, Fernanda Oliveira, Maria Aparecida da Silva, Ursula Matte, Maria S. Pombo-de-Oliveira, Rosane Bittencourt, Liane Daudt, Lúcia Silla

**Affiliations:** ^1^Cellular Therapy Center, Center for Experimental Research, Hospital de Clinicas de Porto Alegre, 90035-903 Porto Alegre, RS, Brazil; ^2^Postgraduate Course of Medical Sciences, Federal University of Rio Grande do Sul, 90035-903 Porto Alegre, RS, Brazil; ^3^Gene Therapy Center, Center for Experimental Research, Hospital de Clinicas de Porto Alegre, 90035-903 Porto Alegre, RS, Brazil; ^4^Pediatric Hematology and Oncology Program, Research Center, Instituto Nacional de Câncer, 20230-130 Rio de Janeiro, RJ, Brazil; ^5^Hematology and Bone Marrow Transplantation, Hospital de Clinicas de Porto Alegre, 90035-903 Porto Alegre, RS, Brazil; ^6^Laboratory of Cell Culture and Molecular Analysis of Hematopoietic Cells, Center for Experimental Research, Hospital de Clínicas de Porto Alegre, 2350 Ramiro Barcelos, 90035-903 Porto Alegre, RS, Brazil

## Abstract

Acute myeloid leukemia (AML) is a complex and heterogeneous hematopoietic tissue neoplasm. Several molecular markers have been described that help to classify AML patients into risk groups. DNA methyltransferase 3A (*DNMT3A*) gene mutations have been recently identified in about 22% of AML patients and associated with poor prognosis as an independent risk factor. Our aims were to determine the frequency of somatic mutations in the gene *DNMT3A* and major chromosomal translocations in a sample of patients with AML. We investigated in 82 samples of bone marrow from patients with AML for somatic mutations in *DNMT3A* gene by sequencing and sought major fusion transcripts by RT-PCR. We found mutations in the *DNMT3A* gene in 6 patients (8%); 3 were type R882H. We found fusion transcripts in 19 patients, namely, AML1/ETO (*n* = 5; 6.1%), PML/RAR**α** (*n* = 12; 14.6%), MLL/AF9 (0; 0%), and CBF**β**/MYH11 (*n* = 2; 2.4%). The identification of recurrent mutations in the *DNMT3A* gene and their possible prognostic implications can be a valuable tool for making treatment decisions. This is the first study on the presence of somatic mutations of the *DNMT3A* gene in patients with AML in Brazil. The frequency of these mutations suggests a possible ethnogeographic variation.

## 1. Introduction

Acute myeloid leukemia (AML) is a complex and heterogeneous hematopoietic tissue neoplasm caused by gene mutations, chromosomal rearrangements, deregulation of gene expression, and epigenetic modifications. These changes lead to unregulated proliferation and loss of differentiation capacity of myeloid hematopoietic cells. In recent years, several important prognostic molecular markers have been described for AML which not only improved disease characterization, but also allowed stratification of patients into risk groups and can guide therapeutic decision-making [1]. However, these molecular markers are often unable to provide accurate prognostic and therapeutic information, since the course of the disease varies significantly between patients belonging to the same risk category [2–4].

The traditional view of cancer as a disease caused by some genetic mutation has been replaced by the concept of a complex network of gene deregulation and epigenetic changes. Additionally, although extremely important, those mutations that have been reported are found in only a minority of patients with AML [5–7]. The distinct components of epigenetic machinery such as DNA methylation, covalent modifications of histones, and noncoding RNAs have been described as cocontrollers of gene expression and within a context of cancer may contribute to leukemogenesis [8]. Methyltransferases such as *DNMT1*, *DNMT3A*, and *DNMT3B* are key components of the epigenetic regulation of genes as they catalyze the addition of methyl groups to the cytosine residue of CpG dinucleotides.

Recently, in a study using whole genome sequencing, recurrent somatic mutations have been described in the DNA methyltransferase 3A gene (*DNMT3A*) in 22% of patients with AML [9]. In this study, *DNMT3A* mutations were independently associated with a poor prognosis and more frequent in patients with normal cytogenetics and as such, of utmost clinical relevance. Eighteen different mutations were found, most of them missense mutations. Preliminary data show that the incidence of these mutations in AML ranges from 4.1% in a Japanese study [10], 9% in a study with Chinese patients [11], and about 15–25% in two Western studies [9, 12–14]. Given the association with CN-AML observed in all studies, it is not astonishing that the highest prevalence was reported in the 2 series focusing on CN-AML (29–36%) [15, 16]. These possible ethnogeographic differences in the incidence of *DNMT3A* mutations as well as their prognostic role need, however, to be better characterized. The exact mechanisms by which of *DNMT3A* mutations act in AML are still unclear, since the global pattern of methylation in the genome of such patients with AML does not appear to be significantly changed [9].

The aim of this study was to characterize the frequency and clinical impact of mutations in the *DNMT3A* gene, correlating it with clinical data and with already well defined translocations in AML in a group of patients treated at the Hospital das Clinicas, in Porto Alegre, Rio Grande do Sul, Brazil.

## 2. Materials and Methods

### 2.1. Patients

 We have studied 87 samples of bone marrow from patients with AML, at diagnosis and prior to any chemotherapy, which had been cryopreserved at the Laboratory of Cell Culture and Molecular Analysis of Hematopoietic Cells belonging to the Center for Experimental Research at the Hospital de Clinicas of Porto Alegre (CPE-HCPA) since 2001 to the present date. The patients' clinical information was obtained from the AML database of the Service of Hematology and Bone Marrow Transplantation of HCPA. Patients were stratified into risk groups—favorable, intermediate, and high—according to the WHO criteria [17]. The favorable subgroup is represented by recurrent reciprocal translocations t(15;17), t(8;21),and inv(16); the intermediate includes patients with a normal karyotype, +8 and t(9;11); and the unfavorablesubgroupincludescomplex karyotypes (≥3 abnormality) −5 and −7 abnormalities, anomalies of chromosome 3, and balanced structural rearrangements as: t(6;9), t(6;11), and t(11;19). Karyotypic characterization of our sample is shown in Table 1.

The procedures were approved by the Ethical Committee of Human Experimentation in Brazil, and are in accordance with the Helsinki Declaration of 1975.

### 2.2. Extraction of DNA and RNA

Samples of cryopreserved bone marrow were thawed, washed with PBS1x with 5% albumin, and then had their DNA and RNA extracted with Trizol Reagent (Invitrogen), according to the manufacturer's recommendations.

### 2.3. Identification of Fusion Transcripts

 After RNA extraction we proceeded to reverse transcription using the *SuperScript III* kit (Invitrogen). The effectiveness of RNA extraction and of cDNA synthesis was monitored by the amplification of the constitutive gene glyceraldehyde-3-phosphate dehydrogenase (*GAPDH*) and negative samples were discarded.

The sequences of interest were amplified by the polymerase chain reaction (PCR) according to BIOMED-1 [18] (Table 2). PCR products were visualized by electrophoresis on 1.5% agarose gel and bands were considered positive in the following sizes: *AML-A/ETO-B*: 395 bp, *PML-A1/RAR*α*-B*: 381 bp, *PML-A2/RAR*α*-B*: 376 bp, *CBF*β*-A/MYH11-B2*: 418, and *MLL6S/AF9AS3*: 651 bp [18, 19].

### 2.4. Identification of Mutations in *DNMT3A* Gene

The extracted DNA was amplified by PCR at the *DNMT3A* exons 19, 20, 21, 22, and 23, with primers described by Thol et al. [14] (Table 2). After electrophoresis on 1.5 agarose gel, PCR products were subjected to purification using Exonuclease I and Shrimp Alkaline Phosphatase (EXO-SAP, GE Healthcare) and then sequenced. 

### 2.5. Sequencing

Samples were sequenced at the Unidade de Análises Moleculares e de Proteínas (Centro de Pesquisa Experimental, HCPA) using ABI 3500 Genetic Analyzer with 50 cm capillaries and POP7 polymer (Applied Biosystems). PCR products were labeled with 3.2 pmol of the forward primer and 1 *μ*L of BigDye Terminator v3.1 Cycle Sequencing Kit (Applied Biosystems) in a final volume of 10 *μ*L. Labeled samples were purified using BigDye XTerminator Purification Kit (Applied Biosystems) and electroinjected in the automatic sequencer. Electropherograms were compared to the reference sequence (NM_022552). Altered sequencing results were confirmed by reverse strand sequencing. 

### 2.6. Statistical Analysis

Statistical analysis was performed using SPSS V18. Overall Survival and Disease-Free Survival curves were calculated using the *Kaplan-Meier* survival function and comparison by the *Long Rank* test. For categorical data *Fisher*'s exact test was used. *P* value of less than 0.05 was considered statistically significant.

## 3. Results

### 3.1. Characterization of the Sample

Of the 87 AML samples taken from the cell bank of the Laboratory of Cell Culture and Molecular Analysis of Hematopoietic Cell, 82 could be analyzed. Of the studied patient population, 58.5% (48) were male with a median age of 42 years. According to the FAB classification, 6.8% (5) were AML M0, 21.9% (16) AML M1, 30.1% (22) AML M2, 19.2% (14) AML M3, 17.8% (13) AML M4, 1.4% (1) AML M5, and 2.7% (2) were classified as AML and not M3. The median white blood cell (WBC) count at diagnosis was 6.6 × 10^9^/L ranging from 0.16 to 374.5 × 10^9^/L. There were 23 (41.8%) cases with karyotype alterations. As for risk stratification, 18 (29%) patients were allocated to the favorable group, 38 (61.3%) to the intermediate group, 6 (9.7%) belonged to the unfavorable risk group, and in 20 (16.4%) karyotypic analysis was not performed and therefore could not be classified (Table 3).

**Figure 1:**
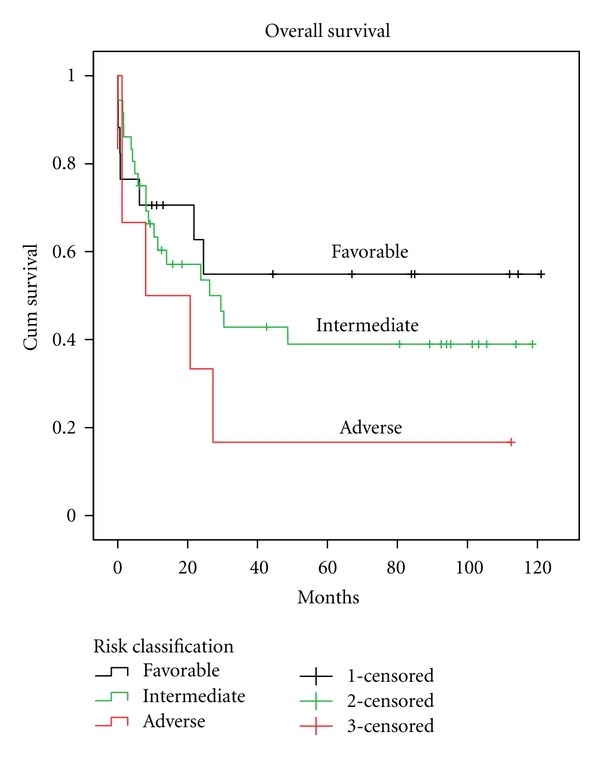
Comparison of the estimated overall survival according to the risk category with a followup of 120 months: favorable (54.9%), intermediate (39%), and unfavorable (16.7%) (*P* = 0.15).

**Figure 2:**
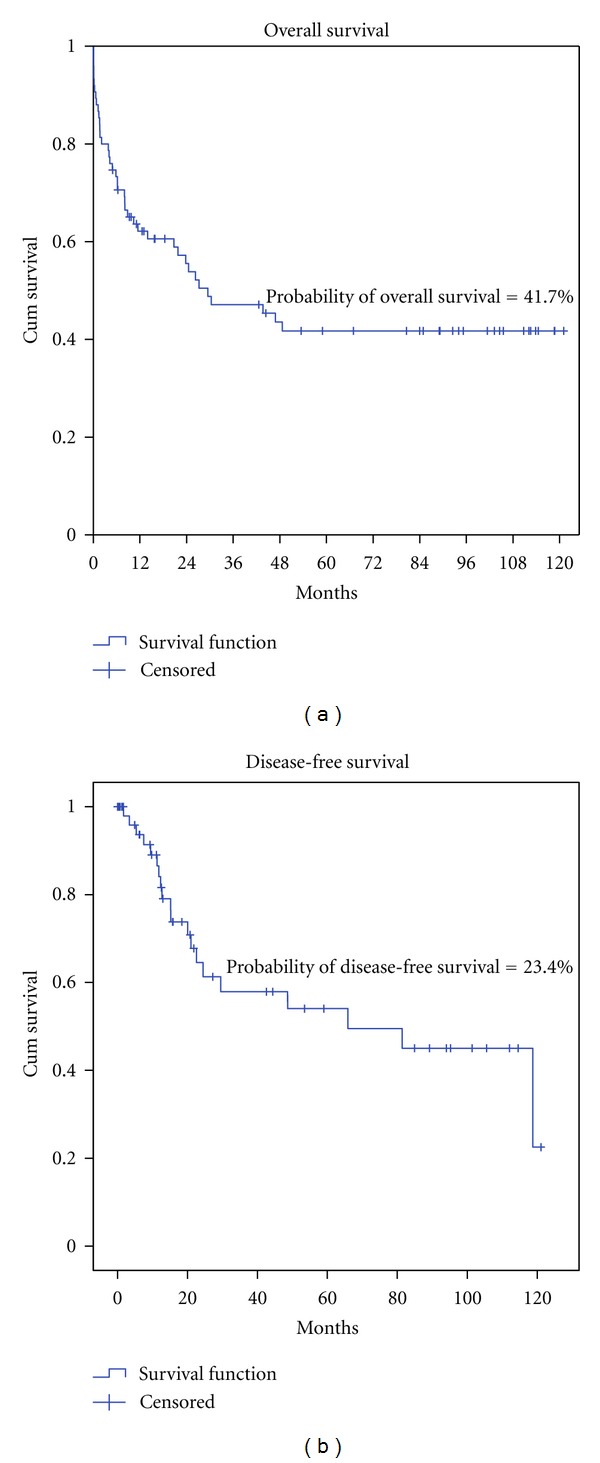
(a) Overall survival in a followup of 120 months with an estimated probability of overall survival of 41.7%; (b) disease-free survival in a followup of 120 months with an estimated probability of disease-free survival of 23.4%.

As shown in Table 3, we were able to stratify into risk categories only 62 patients since for 20 of them we did not have enough information. Eighteen (29.0%) were in the favorable, 38 (61.3%) in the intermediate, and 6 (9.7%) in the unfavorable risk group. Except for the group of patients with AML M3 who were treated according to the APL protocol [20], all other patients received remission induction and consolidation using the protocol 7 + 3, and intensification with high doses of AraC. Of these, 8 were subsequently submitted to autologous and 22 to allogeneic bone marrow transplantation (BMT). Of the entire group, 14 (19.2%) were refractory to treatment. Of these, 1 (7.1%) belonged to the favorable, 8 (57.1%) to the intermediate, 2 (14.2%) to the unfavorable, and 3 (21.4%) belonged to the unclassified group. The overall survival (OS) of the 62 categorized patients, with a followup of 120 months, was 54.9%, 39.0%, and 16.7% for favorable, intermediate, and unfavorable risk category, respectively (*P* = 0.15) (Figure 1). The OS and disease-free survival (DFS) of the entire group of patients, with a followup of 120 months, was 41.7% and 23.4%, respectively (Figure 2).

### 3.2. Fusion Transcripts

Nineteen patients (23.1%) had fusion transcripts identified by RT-PCR. Five (6.1%) presented the *AML1/ETO*, 12 (14.6%) *PML/RAR*α**, and 2 (2.4%) the *CBF*β*/MYH11* fusion genes. The presence of *MLL/AF9* t(9;11) was not found in our series of AML patients. The transcript *PML/RAR*α** was identified in 78.5% (11) of the cases classified as APL. Of the 12 *PML/RAR*α** positive patients, only 4 had a compatible karyotype, positive for t(15;17), and the remaining had either normal (3) or no karyotype (5).

When comparing the overall survival for positive and negative *PML/RAR*α** patients, with a followup of 120 months, we observed that the OS was 72.7% for positive and 37.6% for the negative (*P* = 0.19) (Figure 1). A tendency for prognostic value was also shown for the presence of AML1/ETO with an OS of 22.7% and 60.2% for positive and negative, respectively (*P* = 0.19). Finally, one of the inv16 patients died during remission induction and the other is still alive in continuous complete remission.

### 3.3. *DNMT3A*

**Figure 3:**
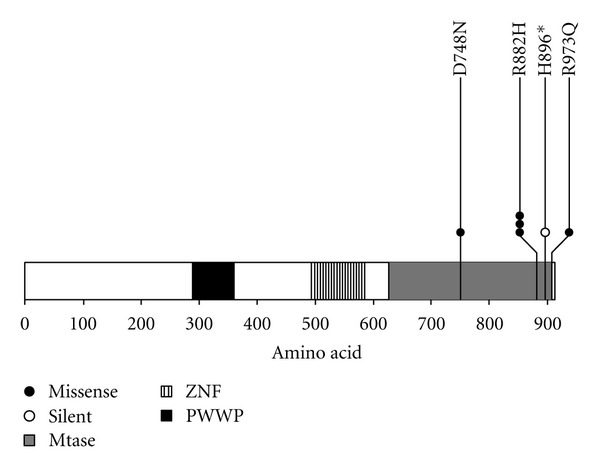
Location and classification of gene mutations found in gene *DNMT3A*. Representation of the *DNMT3A* gene and its domains: *methyltransferase* (MTase), *zinc-finger *(ZNF), and *conserved proline-tryptophan-tryptophan-proline* (PWWP).

Somatic mutations were found in 8% (6) of the samples, being 5 missensemutations and one silent mutation, including the p.R882H mutation described by Ley et al. [9] that was identified in 3 patients. All variant sequences were heterozygous and no patient had more than one mutation. The new mutations found were: p.R973Q, p.D748N, and p.H896. The mutations location domains are shown in Figure 3. Of the 6 cases with *DNMT3A* mutations, the majority (5, or 83.3%) were located in exon 23. Four (80.0%) patients with mutations belonged to the intermediate risk group with normal karyotype, 1 to the favorable group, and 1 unclassified. Of the patients with *DNMT3A* mutation, only 1 was positive for the fusion transcript *PML/RAR*α** and died of coagulopathy during induction; the patient with trisomy 4 and 8 is alive in continuous remission (Table 4).

The characteristics of patients with or without *DNMT3A* gene mutation did not differ significantly, and they are represented in Table 5. Although the sample size does not allow a comparative analysis of survival, with a followup of 120 months, OS for patients with wild *DNMT3A* gene was 41.4% and for patients with mutated *DNMT3A* was 44.4%  (*P* = 0.59); the SLD was 22.7% and 0%, respectively (*P* = 0.32).

## 4. Discussion

Of the 82 patients studied, we were able to classify 73 according to the FAB classification. The frequency of FAB subtypes M0, M1, and M2 was similar to that reported in the literature except for subtypes M4, M5, M6, and M7 whose frequency was lower (Table 3). The M3 subtype was more frequent (19.2%) in our group when compared with international studies; this confirms the results reported by Capra et al. [21] in a study in Rio Grande do Sul, Brazil and is similar to that reported by others for the Latin American population [22, 23]. The frequency distribution of FAB classification subtypes we found in our sample was the same described in 532 AML cases we reported [21] in the same region with patients with the same ethnic background. Based on this finding we can say that although now reporting a smaller sample of patients from a single institution, it is a representative of our population. Our institution is a university public hospital with one of the most active bone marrow transplantation centers in the country to where AML patients from all over the state are referred for treatment. Regarding the classification of risk found in our sample of 29.0%, 61.3%, and 9.7% for the favorable, intermediate, and unfavorable risk categories, respectively, in spite of having a significant number of cases not classified, in general it agrees with the distribution described in the literature and is virtually identical to that reported in patients in the same region of the country [21].

The frequency of fusion transcripts, particularly the *AML1/ETO* found in 6.1% of our sample, was similar to that described in the literature (6 a 12%) [24, 25], while the relative frequency of *PML/RAR*α** (14.6%) was higher (5–8%) [26], probably reflecting the higher incidence of AML M3 in our population. For the transcript *CBF*β*/MYH11* we had a relative frequency of 2.4%, slightly lower than the (5–8%) reported by others [27], and none positive for the transcript *MLL/AF9*, which correlates with the literature, which indicates a frequency of approximately 1% [19]. However, in general, the finding of rearrangements in 22% of our patients is consistent with the frequency of 20% found in 1065 patients in the UK [28]. Finally, the analysis of chromosomal translocations by RT-PCR proved to be advantageous in our center since only 7 of the 19 patients with fusion transcripts were detected by karyotype analysis, explaining the frequency of only 41.8% of karyotype abnormalities found in our group of patients, less than the 65% reported by Look [29].

The search for recurrent somatic mutations in the gene DNA methyltransferase 3A (*DNMT3A*) was performed in all our 82 patients. We chose to sequence the last five exons of the gene *DNMT3A* since, as demonstrated by Ley et al. [9], approximately 80% of the mutations were located in these exons, with 58% of them in the last one (exon 23), where, in fact, most mutations in our study were found (Figure 3). The frequency of somatic *DNMT3A* mutations found in 8% of our 82 cases is lower than the 22% reported in 281 patients by Ley et al. [9], and lower than the 17.8% found by Thol et al. [14], also in Western patients, including about 500 patients. Interestingly, although the first study has sequenced the entire gene, in the latter only the last nine exons were studied. The lowest frequency of mutations in our sample appears similar to that reported for patients of other ethnic groups. In a Japanese study [10], including 74 patients and sequencing the entire gene, the frequency of mutations was found to be only 4.1%, all located in exon 23, while in a Chinese study [11] including 355 patients and also sequencing the entire gene, the frequency of mutations, predominately affecting exon 23, was approximately 9%. 

As for the sequences of *DNMT3A* gene variants, in accordance with a study of Stegelmann et al. [30], all our cases were heterozygous and no patient had more than one mutation; in addition, 3 of 6 mutations were p.R882H, already described by Ley et al. [9] who found a frequency of 59% of such mutation.

Five, or 80%, of our patients harboring a *DNMT3A* mutation belonged to the intermediate risk category, as was reported by others [9]. We also found a tendency (*P* = 0.28) to an increased leukocyte number at diagnosis for patients with mutation (20.7 × 10^9^/L) comparing to the ones without mutation (6.4 × 10^9^/L) which is in agreement with those reported in numerous studies [9–11, 31–33]. Interestingly, and worth mentioning, in our group of patients there was one case of *DNMT3A* mutation that also harbored *PML/RAR*α**.

Finally, the OS according to risk category in our group of 62 patients showed a prognostic trend similar to that reported in the literature (Figure 1). A prognostic evaluation for *DNMT3A* somatic mutations or its concurrency with fusion transcripts could not be determined in our study due to our sample size. 

## 5. Conclusions

In conclusion, to our knowledge, this is the first study on the presence of somatic mutations of the gene *DNMT3A* in patients with AML in Brazil. Although in a small number of patients, we found the frequency of these mutations to be lower than that reported for Western patients. This could indicate an ethnogeographical variation already suggested in the literature for Eastern and Caucasian patients [34]. The discovery of recurrent mutations in the gene *DNMT3A* and its possible prognostic implications can provide valuable information for risk stratification for patients with AML and represents a valuable tool for making therapeutic decisions. However, the use of mutations in the *DNMT3A* gene as a tool for risk stratification needs to be discussed considering their application in different ethnicgeographic groups.

## Figures and Tables

**Table 1:** Karyotypic characterization of our sample.

Result of karyotype result analysis	Number of pts (%)
Normal	38 (61.3%)
t(15;17)	5 (8.0%)
t(8;21)	4 (6.4%)
Complex karyotype	3 (4.8%)
del(11)	1 (1.6%)
del(X)	1 (1.6)%
add(7)	1 (1.6%)
t(6;9)	1 (1.6%)
t(1;2)	1 (1.6%)
t(18;9)	1 (1.6%)
t(3;21)	1 (1.6%)
t(10;11) and del(7)	1 (1.6%)
add(18)(21)(7)	1 (1.6%)
Trisomy 4 and 8	1 (1.6%)
Tetraploid	1 (1.6%)
Polyploidy	1 (1.6%)

**Table 2:** Primer sequences for genes of interest.

Chromosomal translocation	Fusion transcript	Sequence (5′–3′)
t(8;21)	AML1-A	CTACCGCAGCCATGAAGAACC
	ETO-B	AGAGGAAGGCCCATTGCTGAA
		
	PML-A1	CAGTGTACGCCTTCTCCATCA
t(15;17)	PML-A2	CTGCTGGAGGCTGTGGAC
	RAR*α*-B	GCTTGTAGATGCGGGGTAGA
		
inv16	CBF*β*-A	GCAGGCAAGGTATATTTGAAGG
	MYH11-B2	TCCTCTTCTCCTCATTCTGCTC
		
t(9;11)	MLL6S	GCAAACAGAAAAAAGTGGCTCCCCG
	AF9AS3	TCACGATCTGCTGCAGAATGTGTCT
		
Gene	Exon	Sequence (5′–3′)
		
*DNMT3A *	Exon 19	CACCACTGTCCTATGCAGACA
		ATTAGTGAGCTGGCCAAACC
		
*DNMT3A *	Exon 20	CCTTGGCTCATCTTCAAACC
		CACTATGGGTCATCCCACCT
		
*DNMT3A *	Exon 21	CCGCTGTTATCCAGGTTTCT
		CCCAGCAGAGGTTCTAGACG
		
*DNMT3A *	Exon 22	TTTGGTAGACGCATGACCAG
		AGCACAGCAATCAGAACAGC
		
*DNMT3A *	Exon 23	TCCTGCTGTGTGGTTAGACG
		ATGATGTCCAACCCTTTTCG

**Table 3:** Characteristics of the entire patient population.

Variable	Number of patients (%)
Age—*n* = 82	
Median (SD)	42 (18.5)
Mean (SD)	40.6 (18.5)
Range	3–75
Sex—*n* = 82	
Male	58.5% (48)
Female	41.5% (34)
FAB classification—*n* = 73	
M0	6.8% (5)
M1	21.9% (16)
M2	30.1% (22)
M3	19.2% (14)
M4	17.8% (13)
M5	1.4% (1)
M6	0% (0)
M7	0% (0)
AML not M3	2.4% (2)
Karyotype—*n* = 55	
Normal	58.2% (32)
With alteration	41.8% (23)
Risk classification—*n* = 62	
Favorable	29% (18)
Intermediate	61.3% (38)
Unfavorable	9.7% (6)
Leukocytes (×10^9^/L)—*n* = 82	
Median (SD)	6.6 (51.9)

**Table 4:** Description of somatic mutations found in gene *DNMT3A*.

Patient identification	Mutation	Allelic change	Exon	Type of mutation	FAB subtype	PCR	Risk group	Karyotype
39	D748N	G>A	19	*Missense *	M1	Negative	Intermediate	Normal
79	R882H	G>A	23	*Missense *	M1	Negative	Intermediate	Trisomy (8)(9)
4	R882H	G>A	23	*Missense *	M3	Negative	Intermediate	Normal
70	R882H	G>A	23	*Missense *	M2	Negative	Intermediate	Normal
41	H896*	A>G	23	*Silent *	M3	PML/RAR*α*	Favorable	t(15;17)
78	R973Q	G>A	23	*Missense *	—	Negative	—	—

**Table 5:** Clinical characteristics of patients with acute myeloid leukemia with or without *DNMT3A* mutations.

Characteristics	Number of pts (%) DNMT3A mutated	Number of pts (%) DNMT3A not mutated	*P*
Age (median)	40.2	44.8	0.56
Sex			
Male	50% (3)	59.3% (45)	0.68
Female	50% (3)	40.7% (31)	
Subtype FAB			
M0	0%	7.2% (5)	
M1	60% (3)	18.8% (13)	
M2	20% (1)	30.4% (21)	0.56
M3	20% (1)	20.3% (14)	
M4	0%	18.8% (13)	
M5	0%	1.4% (1)	
Not M3	0%	2.9% (2)	
Risk groups			
Favorable	20% (1)	29.8% (17)	
Intermediate	80% (4)	59.6% (34)	1.000
Unfavorable	0%	10.5% (6)	
Leukocytes (×10^3^) (median)	20.67	6.41	0.28
Death	50% (3)	51.4% (37)	1.000
Relapses	50% (2)	30.9% (17)	0.58
Refractory	20% (1)	19.1% (13)	0.96
